# Answer to Wang and Luo, "Polyploidization increases meiotic recombination frequency in *Arabidopsis*: a close look at statistical modelling and data analysis"

**DOI:** 10.1186/1741-7007-10-31

**Published:** 2012-04-18

**Authors:** Ales Pecinka, Avraham Levy, Ortrun Mittelsten Scheid

**Affiliations:** 1 Max Planck Institute for Plant Breeding Research, 50829 Cologne, Germany; 2 Department of Plant Sciences, Weizmann Institute of Science, 76100 Rehovot, Israel; 3 Gregor Mendel Institute of Molecular Plant Biology, 1030 Vienna, Austria

## Abstract

This article is a response to Wang and Luo.

See correspondence article http://www.biomedcentral.com/1741-7007/10/30/ [WEBCITE] and the original research article http://www.biomedcentral.com/1741-7007/9/24 [WEBCITE].

## Background

Meiosis is an obligate process during sexual reproduction that involves the combination of parental genomes and the coordinated segregation of the recombined chromosomes to the gametes. Polyploidy, the presence of more than two sets of chromosomes per nucleus, has direct and fundamental consequences on meiosis, which are gradually and individually different between the extreme cases of auto- and allopolyploids (multiplied chromosome sets or combination of slightly different chromosome sets). Polyploidy has a major impact on the segregation of genotypes and phenotypes in progeny. In a recent study [1], we described that polyploidy increased the frequency of meiotic recombination between two genetically linked transgenes providing seed-specific fluorescence. This increase was seen in reciprocal crosses of genetically identical diploid and autotetraploid *Arabidopsis thaliana*, but also in reconstituted hybrids resembling allotetraploid *A. suecica*. In a comment, Wang and Luo [2] question the validity of the data analysis after subjecting the experimental data to a different calculation method.

## Response

It is correct that genetic segregation analysis in autotetraploids requires considering the possibility of multivalent formation during pachytene and a potential double reduction of genetic loci. Both special conditions of autotetraploid meiosis are mentioned in [1], p. 1-2. We agree that a more detailed tetrasomic linkage analysis could improve the data analysis for segregation in autotetraploids. We lack the mathematical expertise to critically review the different theoretical models presented in [2] and refer to a detailed comment in [3]. However, the evaluation of meiotic recombination suggested by [2] also results in significantly higher rates in auto- and allopolyploids on one side and diploids on the other. Therefore, the claim in [2] for 'qualitative differences from the original analysis' is not justified.

Further, the statement that we 'concluded that meiotic recombination was more frequent in the allotetraploids than in the autotetraploids' is incorrect: we wrote that the increase of recombination frequency of both polyploids over diploids was 'in the same range' (p. 4), which holds true for both different evaluations [1,2].

Third, the green marker on the tester chromosome is further (distal) from the centromere [1,4], not 'nearer to the centromere than the red marker' as assumed in [2].

We have corrected three values in Table 1 according to the comments in [3].

In summary, we are confident that the conclusions from our work remain unchanged.
